# Therapeutical and Pharmaceutical Notes

**Published:** 1900-08

**Authors:** W. J. Martin

**Affiliations:** Kankakee, Ill.


### THERAPEUTICAL AND PHARMACEUTICAL NOTES.

UNDER THE DIRECTION OF W. J. MARTIN, M.D.C.,
KANKAKEE, ILL.

OIL OF TURPENTINE AS A THERAPEUTIC AGENT. Oil of turpentine being an agent much used in veterinary practice, we give the following symposium of the drug for what it is worth:

In tetanus: Dr. Cooper, the editor of the *Eclectic Medical Gleaner*, is not a believer in serum-therapy. Speaking of the treatment of tetanus lately he gives his own method for what it may be worth, he having treated a dozen cases, three typical, with trismus and opisthotonos, three less developed, and the other six showing only prodromata. All of these were tramautic cases and all of them recovered. He employed the same treatment in all these cases. "I know why I followed this clinical method after my experience with the first case," Dr. Cooper writes; "but I only partially know why I adopted the treatment in the first instance. In several of the cases the wound seemed to be entirely beyond the tetanic symptoms developed. In each case I reopened or freshened the wound, and I kept it saturated at first (for a day or two) with oil of turpentine. After a couple of days I used this more sparingly. I used this remedy because I had often heard Dr. West, an old and experienced physician, say that he had never known a punctured or lacerated wound (or any other kind, of course), which was treated in this way to result in lockjaw. That, too, has been my experience. This would seem to give color to the theory that there is a specific something (possibly a microbe) which causes tetanus. Turpentine is a germicide, but it is also several other things. My other local treatment consisted in having the patient's spine rubbed frequently with strong mustard-water, quite warm. I had the spine kept in an erythematous condition all through. For internal treatment I gave gelsemium and macrotys in combination. I pushed them to the physiological limit. The quantity and frequency of the dose must be controlled by the age, sex, and general physical condition of the patient."

In diphtheria: Dr. W. C. Wright, of Unionville, Mich., contributes a note to the *Post Graduate*, in which he says that he has treated thirty-seven cases of diphtheria without a death or any alarming symptoms. He writes: "To a child from ten to sixteen years old I have given teaspoonful doses of undiluted oil of turpentine every two hours until from four to six doses have been administered, and then wait about forty-eight hours before repeating the dose. In only five cases out of thirty-seven have I had to repeat the medication. In nearly every case the temperature returned to normal inside of twelve hours, and remained so. In two cases the temperature went up a second time, but again returned to normal when the oil of turpentine was given again. The only reason I can suggest in explanation of the absence of any manifestation of urinary disorder followed the administration of such large and frequently-repeated doses of oil of turpentine is that in diphtheria turpentine is an antidote to the toxins as well as an antiseptic.

Production of Camphor. At the present time Japan, by the acquisition of the Island of Formosa, has practically a monopoly in the production and sale of the world's supply of camphor. According to recent United States consular reports Japan proper produces but 300,000 pounds of camphor annually; China never exceeds 220,000 pounds; while the island of Formosa yielded, in 1895, 7,000,000 pounds. Since that period the annual production of camphor in Formosa has averaged about 6,000,000 pounds.

The production and refining of camphor in Formosa has lately passed under the complete control of the Japanese Government, which fixes the amount that shall be annually produced and exported from the island. The price paid to the native camphor producers in the interior of the island by the Government for the crude camphor is about $15 per 133 pounds.

For the present at least, and perhaps for many years to come, camphor is likely to remain at its present high price. Attempts have of late years been made to introduce the camphor tree into the southern part of Florida, and from recent accounts this experiment is meeting with a fair degree of success.

ARSENIC IN ORGANIC BODIES. M. Gautier, after an exhaustive chemical analysis of the human body, finds that arsenic is present in the normal thyroid gland ($\frac{3}{4}$ of a millegramme for each 100 grammes of thyroid-body). Arsenic is also found in the skin, in the hair, in the mammary glands, in the milk, in bone and brain-tissues. No arsenic was found in the following organs: The liver, the kidneys, the muscles, the testes and seminiferous tubules, the mucous membrane, the uterus, and the blood.

A CASE of death after antirabic treatment has just occurred in Paris, and was described by Dr. Menetrier. The only really apparent symptom was hydrophobic spasm. Death took place by syncope. On performing the autopsy no lesion of the nervous system could be found.

BICHLORIDE EYE-WASH. Mercuric chloride, 0.05; rose-water, 150; tinct. of opium, 1.5; mix.

LASSAR'S SUBLIMATE-CARBOLIC SALVE. Mercuric chloride, 0.5 to 1; carbolic acid, 10 to 20; zinc ointment, 5000; mix.

OIL of turpentine is said to remove the odor of iodoform from the hands or instruments.

HEALING OINTMENT. Dr. L. C. Allen, of Hoschton, Ga., writes that he has found the following an effective, all-round, healing-ointment, which at the same time is cheap. Its beneficial action is due to the antiseptic and stimulating properties of the oil of turpentine. Melt together and stir until cold, beeswax, 1 ounce; petrolatum, 1 ounce; oil turpentine, 1 ounce.

ANTISEPTIC WOUND OIL. The same correspondent is fond of using the following dressing in minor surgery, such as cuts, wounds, after removal of tumors, etc.: Oil of turpentine, 1 ounce; olive oil, sterilized, 1 ounce. A liberal quantity of this is poured over the wound, and a thin pad of gauze saturated with it is laid on. Over this more gauze (cotton), then bandage. The oil keeps the surface of the wound moist, allowing the liquid excretions to escape from the wound (drainage) and be carried off into the dressing by capillary attraction. This preparation is a powerful stimulant and antiseptic, and wounds heal rapidly and aseptically under its use.

Salt an Antidote to Strychnine. The following interesting editorial note appeared in a late issue of the *Alkaloidal Clinic:* An observant friend tells me that he has frequently seen dogs saved from strychnine-poisoning by the use of large doses of common salt; this after the dog was in spasms. He first saw some boys recovering a dog that had been treated to a dose of strychnine by the police. The method used was to fill the dog's mouth with a big handful of salt and wash it down with water from an old tin can. The dog soon straightened out and was all right. Is it a chemical antidote, or does it cause elimination of the poison by creating an active exosmosis? Who knows? Who has made the same observation? Does it apply to the human family?—*Western Druggist.*

Creolin Soap. Tonzig (*Gazzeta degli Ospedali*, January 14, 1900) has made a series of experiments with creolin soaps of various strengths and manufacture. Most of the common disinfecting soaps have been found by experimenters to be less efficacious for purposes of disinfection than the simple soaps. With regard to the creolin soaps the author tested their germicidal power on a species of chlorigenic vibrio and found that with one single exception (a resinous soap containing 20 per cent. of creolin) they were all less active as germicides than simple soaps of the same nature, but minus creolin. Five different kinds of soap containing creolin in various proportions were tried. In the author's opinion the chief benefit in adding creolin to the soap is derived by the manufacturer, for it adds nothing to, but detracts from the disinfecting power of the soap.—*British Medical Journal, February 24, 1900.*

